# Elevated ROS levels during the early development of Angelman syndrome alter the apoptotic capacity of the developing neural precursor cells

**DOI:** 10.1038/s41380-023-02038-7

**Published:** 2023-03-29

**Authors:** Lilach Simchi, Pooja Kri Gupta, Yonatan Feuermann, Hanoch Kaphzan

**Affiliations:** Sagol Department of Neurobiology, Faculty of Natural Sciences, University of Haifa, Haifa, Israel

**Keywords:** Neuroscience, Cell biology

## Abstract

Angelman syndrome (AS) is a rare genetic neurodevelopmental disorder caused by the maternally inherited loss of function of the *UBE3A* gene. AS is characterized by a developmental delay, lack of speech, motor dysfunction, epilepsy, autistic features, happy demeanor, and intellectual disability. While the cellular roles of UBE3A are not fully understood, studies suggest that the lack of UBE3A function is associated with elevated levels of reactive oxygen species (ROS). Despite the accumulating evidence emphasizing the importance of ROS during early brain development and its involvement in different neurodevelopmental disorders, up to date, the levels of ROS in AS neural precursor cells (NPCs) and the consequences on AS embryonic neural development have not been elucidated. In this study we show multifaceted mitochondrial aberration in AS brain-derived embryonic NPCs, which exhibit elevated mitochondrial membrane potential (ΔΨm), lower levels of endogenous reduced glutathione, excessive mitochondrial ROS (mROS) levels, and increased apoptosis compared to wild-type (WT) littermates. In addition, we report that glutathione replenishment by glutathione-reduced ethyl ester (GSH-EE) corrects the excessive mROS levels and attenuates the enhanced apoptosis in AS NPCs. Studying the glutathione redox imbalance and mitochondrial abnormalities in embryonic AS NPCs provides an essential insight into the involvement of UBE3A in early neural development, information that can serve as a powerful avenue towards a broader view of AS pathogenesis. Moreover, since mitochondrial dysfunction and elevated ROS levels were associated with other neurodevelopmental disorders, the findings herein suggest some potential shared underlying mechanisms for these disorders as well.

## Introduction

Neurodevelopmental disorders (NDDs) are a group of neuropsychiatric conditions resulting from developmental impairments in the central nervous system. NDDs are associated with multifaceted abnormalities such as developmental delay and cognitive and behavioral deficits that impose a substantial health burden [1]. Angelman syndrome (AS) is a rare genetic neurodevelopmental disorder occurring in ~1:15,000 live births, which is characterized by a developmental delay, lack of speech, motor dysfunction, epilepsy, happy demeanor, and intellectual disability [2]. Like with many other neurodevelopmental disorders, up to date, there are no therapeutic approaches that address either the prevention of AS or attenuating the development of AS-related symptoms before appearance. Moreover, currently, the available treatments for AS patients aim to treat related symptoms after AS has been diagnosed.

The etiology for AS is the loss of function of the neuronal *UBE3A* gene, which encodes a ubiquitin ligase E3A (UBE3A) enzyme, also termed E6-associated protein, that polyubiquitinates its substrate proteins, promoting them to their degradation by the proteasome system [3]. In neurons, UBE3A expression is epigenetically modified so that transcription of paternal UBE3A is silenced after birth, and only the maternally-inherited copy is expressed. Since AS is caused by the deletion or a mutation of the maternal *UBE3A* gene, the expression of a functioning UBE3A during AS embryonic development is significantly reduced and becomes almost absent once the paternal *UBE3A* gene is silenced [4].

Since the discovery of the genetic etiology of AS, extensive efforts have been devoted to the understanding of the underlying mechanisms of AS pathophysiology, mainly by utilizing the AS mouse model, which was shown to replicate multiple phenotypes of the human disorder [5, 6]. Nonetheless, although a few UBE3A target proteins have been identified, none of these substrates were directly linked to AS phenotypes in mice or humans [7]. Furthermore, up to date, little is known about the downstream molecular mechanisms governed by UBE3A during embryonic development and after.

Recent studies suggested that UBE3A deficiency alters mitochondrial functioning resulting in oxidative stress [8–12], thus contributing to the deleterious effects on brain functionality [12]. In particular, these studies pointed to mitochondrial involvement and oxidative stress in the hippocampus, one of the prominently affected brain regions in AS model mice [13]. Furthermore, studies that examined the AS adult hippocampus showed aberrant mitochondrial morphology, decreased oxidative phosphorylation complex-III activity [10], and elevated reactive oxygen species (ROS) levels in CA1 neurons [12]. In addition, one of these studies demonstrated that administering a mitochondrial antioxidant that normalize ROS levels in the AS hippocampal CA1 neurons rescue the hippocampal synaptic plasticity deficits and the contextual fear memory deficits exhibited by AS mice [12].

The implication of mitochondria in AS pathophysiology is not entirely surprising since mitochondria serve as the hub of multiple critical cellular processes that affect cell viability and functionality beyond ATP production, such as calcium signaling, apoptosis, and neuronal excitability [14–19]. In addition, the mitochondria are the primary source of ROS production, which play a significant role in regulating normal physiological functions in development, such as proliferation, differentiation, and apoptosis [20–24].

Most animal-based studies of neurodevelopmental disorders, including AS, have primarily focused on adult animals. However, recent studies of *Ube3a*-reinstatement highlighted the need to reposition the focus of AS investigation toward early brain developmental stages [25–27]. In these studies, the researchers have attempted to pursue the critical time period for the AS intervention by a conditional *Ube3a*-reinstatement along different developmental time points. These studies showed that the autistic traits of AS phenotype were impervious to rescue by postnatal reinstatement, whereas only embryonic reinstatement resulted in a complete phenotypical rescue [25–28].

Another study by us that investigated the effects of *Ube3a* in the embryonic stage showed that complete deletion of *Ube3a* (*Ube3a*^p−/m−^) altered the mRNA expression and the proteomic profiles of mitochondrial and oxidative stress-related pathways in mouse embryonic fibroblasts (MEFs). We also demonstrated that this complete *Ube3a* deletion in MEFs affected the proliferation and apoptosis capacity of these cells [29, 30].

Based on the above, we predicted that the cellular phenotype of elevated ROS levels and altered mitochondrial function observed in the mature AS brain is not restricted to the adult stage when the AS autistic symptoms are manifested, but appear earlier during neonatal brain development. Moreover, since ROS are known to have a significant role in signaling pathways and regulating physiological functions during development [20], we aimed to study the consequences of elevated ROS levels during AS embryonic neuronal development.

For this we utilized AS embryonic neural precursor cells (NPCs), and demonstrated that during AS embryonic development there is an increase in mitochondrial membrane potential, elevated ROS levels and enhanced apoptosis. We also identified that aberrations in the glutathione system is a primary suspect for the elevated ROS levels phenotype and demonstrated that by replenishing reduced glutathione levels, we reduced mROS expression to normal levels and rescued the phenotype of enhanced apoptosis.

This study addresses, for the first time, the involvement of UBE3A in mitochondrial function during the embryonic development of Angelman syndrome, and the critical role of ROS in controlling the fate of neural stem and progenitor cells in AS brain development. Moreover, based on the accumulating evidence that associates mitochondrial abnormalities and elevated ROS levels with multiple neurodevelopmental disorders such as autism [31–34], schizophrenia [35], Rett syndrome [36], Down syndrome [37], and others [20, 38], it is possible that the herein findings carry significance to the early development of these neurodevelopmental disorders as well, and that mitochondrial anomalies and elevated ROS levels during embryonic development are part of their etiology.

## Materials and methods

### Mice and monolayer neural precursor cells (NPCs) culture preparation

Heterozygous mice with maternal *Ube3a* deletion were generated on a C57BL/6 background and genotyped using specific primers, as described previously [5]. For NPCs culture generation, a heterozygous female mouse with paternally derived *Ube3a* deletion (*Ube3a*^p−/m+^) and a wild-type (WT) (*Ube3a*^p+/m+^) male were bred to generate WT (*Ube3a*^p+/m+^) and AS (*Ube3a*^p+/m−^) littermates. On gestational day E16.5, we extracted the entire brains of the AS and WT littermates’ embryos and dissociated them into a single-cell suspension using Neural Tissue Dissociation Kit (Miltenyi Biotec #130-093-231). Cells were collected by centrifugation (300 × *g* for 5 min) and re-suspended in a complete embryonic NeuroCult™ proliferation medium (STEMCELL #05702) with added EGF (10 µg/ml; STEMCELL *#*78006.1). Next, we plated the cells with the above medium for NPCs expansion in adherent monolayer culture (supplementary Fig. 1). The NPCs were cultured in poly-D-lysine (PDL, 10 µg/ml; Sigma-Aldrich *#*P7280) and laminin (10 µg/ml; Sigma-Aldrich *#*L2020) coated 25 cm^2^ rectangular canted neck flasks with vented cap (Corning *#*353108 in an 8 × 10^4^ cells/cm^2^ density) at 37 °C with 5% CO_2_. Cells were detached with ACCUTASE (STEMCELL #07922) for cell passaging and then seeded in 25 cm^2^ flasks or flat bottom 96-well plates, according to the experimental requirements. We used NPCs within the first to the fifth passage in this study. All samples were biological replicates. Experiments were replicated at least three times. All the mice used for breeding were kept on a 12 h light/dark cycle and had access to food and water ad libitum. Housing, handling, and experimental procedures were approved by the University of Haifa Institutional Committee for animal experiments in accordance with National Institutes of Health guidelines.

### Cellular treatment for glutathione replenishment

Glutathione-reduced ethyl ester (GSH-EE; Sigma-Aldrich *#*G1404) was used for pharmacological replenishment of GSH in WT and AS cultured NPCs. A stock solution of 100 mM in sterile water was prepared (stored at −20 °C) and then further diluted to 0.5 mM working solution (final con.) in a complete embryonic NeuroCult™ Proliferation medium before the treatment. For GSH-EE treatment, the medium was replaced by the working solution for 48 h incubation. As a control, for the separate cohorts of WT and AS vehicle NPCs, the medium was replaced with fresh medium without the supplement of GSH-EE.

### Cellular treatment for glutathione depletion

L-Buthionine-Sulfoximine (BSO; Sigma-Aldrich *#*B2515) was used for pharmacological glutathione depletion in WT NPCs. A stock concentration of 0.22 M in sterile water was prepared and then diluted to the final concentration (see below) in a complete embryonic NeuroCult™ Proliferation medium prior to the beginning of the treatment. For analysis of total glutathione levels by GSH/GSSG-Glo^TM^ (Promega #V6611), the medium was replaced by a 200 µM working solution for 5 h incubation. For TUNEL and Annexin-V/PI assays, the medium was replaced with a 100 µM working solution for 20 h and 4 h, respectively. For each experiment, as a control, we replaced the medium to separate cohorts of WT vehicle NPCs with a fresh medium without the supplement of BSO.

### Western blot analysis

Western blot analysis was carried out for AS (*Ube3a*^p+/m−^) and WT (*Ube3a*^p+/m+^). The cells were lysed with an ice-cold lysis buffer containing: 10 mM HEPES pH 7.5 (Sigma-Aldrich #H4034), 150 mM NaCl (Sigma-Aldrich #71376), 50 mM NaF (Sigma-Aldrich #201154), 1 mM EDTA (Sigma-Aldrich *#*ED), 1 mM EGTA, 10 mM Na_4_P_2_O_7_ (Sigma-Aldrich #P8010) PMSF, and protease inhibitor cocktail (Roche #11873580001). Cell lysates were loaded (15–20 µg per lane) to 4–20% TEO TRICINE gradient gels (abcam ab119209) and transferred to PVDF membranes (abcam ab133411). The membrane was blocked in 5% bovine serum albumin (BSA; Roche #10735086001) in tris-buffered saline with tween20 (TBST) before incubation with primary antibodies for UBE3A (anti-mouse; 1:1000; Sigma-Aldrich #E8655), BCL-2 (anti-rabbit, 1:2000, abcam ab182858), BAX (anti-rabbit, 1:2000, abcam ab182733) overnight. β-ACTIN (anti-mouse; 1:40,000; MP Biomedicals #69100) was used as a loading control. Next, the blots were incubated with the secondary antibodies goat anti-mouse or anti-rabbit IgG (H + L) (1:10000; Jackson *#*115–035–062 and *#*115-035-068, respectively). The bands were detected by chemiluminescence (abcam ab133406) and imaged using the Image Quant LAS 4000 system. All signals were normalized by the total protein and quantified using Image Studio Lite Ver 5.2 software.

### BrdU incorporation and Nestin staining

NPCs were incubated with 10 µM BrdU (BD Biosciences *#*51–2420KC) in a complete embryonic NeuroCult™ proliferation medium. Following 45 min, the NPCs were washed with phosphate-buffered saline (PBS; Sartorius *#*02-023-1A), detached with ACCUTASE, and stained against Nestin (1 µg per 1 × 10^6^ cells, SANTA CRUZ *#*sc-33677PE) and BrdU (BD Biosciences *#*51-51-23619L) using BD Pharmingen™ APC BrdU Flow Kits (BD Biosciences #552598) according to the manufacturer’s protocol. The percentage of BrdU-positive (BrdU^+^) and Nestin-positive (Nestin^+^) NPCs were measured separately by fluorescence-activated cell sorter (FACS; BD FACSCantoII, BD Biosciences) and analyzed with FlowJO_v10.8.1 software.

For cell cycle analysis, a separate experimental cohort of cells was washed with PBS and detached with ACCUTASE after a 45 min BrdU pulse (BioLegend #77528). Then, cells were stained with anti-BrdU antibody and 7-AAD utilizing the Phase-Flow™ BrdU Cell proliferation Kit (BioLegend #370704) according to the manufacturer’s protocol. We monitored the fluorescence labeling by FACS and analyzed it with FlowJO_v10.8.1 software to define the percentage of cells in each cell cycle phase: G0/G1, S-phase, and G2/M (Fig. 2A).

### Immunofluorescence staining

**Fig. 1:**
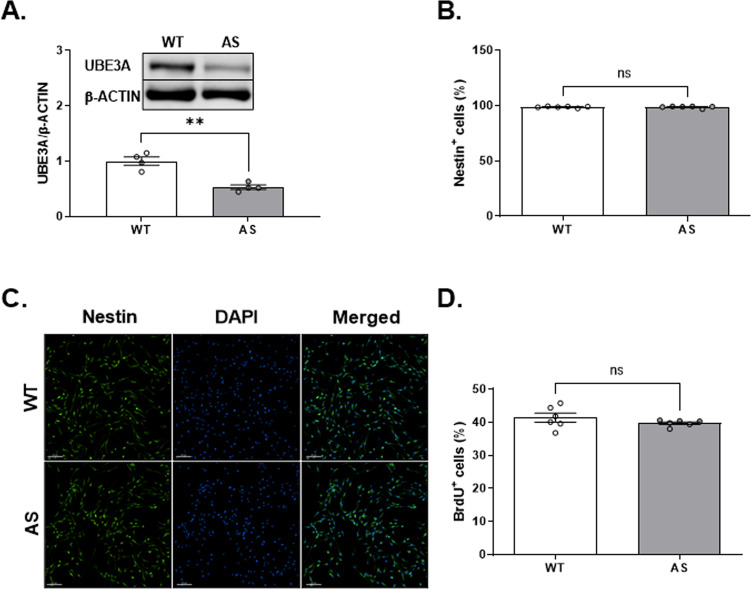
Brain-derived cultured cells from E16.5 mouse embryos in NeuroCult™ proliferation medium are almost all neural precursor cells (NPCs) in both AS and WT littermates. **A** Western blot analysis and representative blots of UBE3A expression in WT and AS NPCs cultured from E16.5 mouse embryonic brains. AS NPCs show ~50% reduction in the levels of UBE3A expression compared to WT. β-ACTIN was used as a loading control. *N* = 4 for each group. **B** FACS analyses demonstrate that more than 98% are Nestin-positive (Nestin^+^) cells. The percentage of Nestin^+^ cells is similar between WT and AS NPCs. *N*  =  6 for each group. **C** Representative immunofluorescence images demonstrate that most cells are stained positive for Nestin. Scale bar, 80 µm. **D** FACS-based analysis of BrdU fluorescence shows an equal percentage (~40%) of BrdU-positive (BrdU^+^) cells in WT and AS cultures. *N*  =  6 for each group. Data are presented as means ± SEM (ns=non-significant, **p < 0.01 in t-test).

Cells were cultured on a coated 13 mm glass coverslip (PDL and laminin, as aforementioned) for 48 h in a complete embryonic NeuroCult™ proliferation medium. The cells were washed with PBS, fixed with 4% paraformaldehyde (MERCK *#*30525-89-4) in PBS for 20 min at room temperature (RT), and subsequently permeabilized with PBS containing 0.5% Triton X-100 for 5 min. Then, the cells were incubated with a blocking solution containing 10% goat serum and 0.2% BSA (Biological Industries Israel Beit Haemek *#*030-010-1B) in PBS with 0.1% Triton X-100, for 1 h at RT, followed by primary antibody incubation with anti-Nestin (anti-rat 1:250, abcam ab81462) at 4 °C overnight. Next, secondary antibody donkey anti-rat 488 (1:500, abcam ab150153) was incubated for 1 h at RT. Washing with 0.1% Triton X-100 in PBS preceded each step. Finally, coverslips were mounted in Fluoroshield™ Histology Mounting Medium with DAPI (Sigma-Aldrich *#*F6057). The representative images were taken with a Nikon Eclipse Ti2 wide-field fluorescence microscope (Fig. 1C).

### TUNEL assay

Apoptosis of NPCs was measured by TUNEL assay kit- BrdU-Red (abcam ab66110), according to the manufacturer’s protocol. Briefly, NPCs were collected and fixed with 4% PFA for 15 min on ice and then were centrifuged and washed with ice-cold PBS. The cells were incubated with 70% ethanol at −20 °C overnight. Next, the cells were incubated in DNA labeling solution for 60 min at 37 °C, followed by sequential 30 min incubations of BrdU antibody solution and 7-AAD at RT. Before each incubation step, the cells were washed with the recommended washing solution according to the manufacturer’s protocol. The percentage of apoptotic TUNEL-positive (TUNEL^+^) cells was detected by FACS and analyzed with FlowJO_v10.8.1 software.

### Annexin-V/PI assay

To investigate the extent of different apoptotic stages, we utilized FITC-conjugated Annexin-V/ Propidium Iodide (PI) assay, using a MEBCYTO Apoptosis Kit (MBL *#*4700). NPCs were detached and collected using ACCUTASE and were washed once with PBS. After centrifugation, the NPCs pellet was re-suspended with binding buffer and incubated with the Annexin-V and PI antibodies mixture for 15 min, according to the manufacturer’s protocol. The fluorescence emissions from the Annexin-V/PI labeled NPCs were then measured by FACS and utilized to discriminate between the different subpopulations: viable cells (Annexin-V^-^/PI^-^), early apoptotic cells (Annexin-V^+^/PI^-^), and late apoptotic cells (Annexin-V^+^/PI^+^) (Fig. 3C). The percentage of each subpopulation was analyzed using FlowJO_v10.8.1 software. To visualize the quality of PI staining of apoptotic cells, we labeled the NPCs with PI and imaged the cells utilizing the CytoSMART Omni FL system (supplementary Fig. 2).

### Luminescence-based assays

Caspase-3/7 and caspase-8 activity, were measured using Caspase-Glo® 3/7 (Promega *#*G8090) and Caspase-Glo® 8 (Promega *#*G8200), respectively. For total glutathione and NADP^+^-NADPH measurements, we utilized GSH/GSSG-Glo^TM^ (Promega #V6611) and NADP/NADPH-Glo^TM^ (Promega *#*G9081), respectively. For each experiment, 5 × 10^4^ NPCs were plated on a coated white 96-well flat bottom plate (Greiner bio-one *#*655098) and left for 12 h of incubation with a complete embryonic NeuroCult™ Proliferation medium at 37 °C with 5% CO_2_. For caspase’s activity measurements in response to GSH-EE treatment, the medium was replaced 12 h after seeding with either medium alone as control or 0.5 mM GSH-EE supplemented medium (vehicle vs. GSH-EE) for 48 h incubation. To measure the total glutathione levels in response to BSO treatment, we replaced the medium 12 h after seeding with either medium alone as control, or 200 µM BSO supplemented medium (vehicle vs. BSO) for 5 h incubation. The experiments were further conducted according to the manufacturer’s protocols. The luminescence signals were measured by Infinite 200 PRO plate reader (Tecan Life Sciences) and analyzed with Tecani-control 1.9.17 software.

All the signals were normalized to the relative number of cells, estimated by CellTiter 96® AQueous One Solution Cell Proliferation Assay (MTS) (Promega # G3582). Briefly, NPCs were cultured in parallel into an additional flat bottom 96-well plate (5 × 10^4^ cells/well in triplicates) with 100 µl complete embryonic NeuroCult™ proliferation medium at 37 °C with 5% CO_2_. The CellTiter 96 AqueousOne Solution (20 µl) was added to each well for 2 h. The absorbance was recorded using a plate reader (Epoch BioTek) at 490 nm and analyzed by Gen5 3.03 (BioTek) software.

### Reduced-glutathione levels analysis

Fluorometric GSH/GSSG Ratio Detection Assay Kit (abcam ab138881) was used to measure the relative GSH levels of WT and AS NPCs in response to GSH-EE treatment. Briefly, samples of WT and AS-treated NPCs and their respective vehicle NPC groups (1 × 10^6^ cells per sample) were prepared with mammalian cell lysis buffer (abcam ab179835) and deproteinizing sample preparation kit -TCA (abcam ab204708) according to the manufacturer’s instructions. Next, 50 µl from each sample was transferred in triplicates to a black 96-well flat bottom plate (Greiner bio-one *#*655090), and 50 µl of GSH assay mixture was added for 30 min incubation at RT. The fluorescence signal was monitored at Ex/Em = 490/520 nm with Synergy H1 Hybrid microplate reader (BioTek) and analyzed by Gen5 3.03 (BioTek) software.

### Mitochondrial membrane potential (ΔΨm) assay

The ΔΨm was measured as previously described [39]. Briefly, cells were dissociated with ACCUTASE and washed with 0.2% BSA in PBS. After centrifugation, the NPCs were incubated for 20 min at 37 °C with the following fluorescent probes: Live/Dead calcein violet-AM (0.9 µM, Invitrogen *#*C34858), tetramethylrhodamine ethyl ester (TMRE)-Mitochondrial Membrane Potential (TMRE; 142.8 nM; abcam ab113852), and Mitochondrial Tracker Green (75.3 nM, abcam ab228568). After a single wash with 5% fetal calf serum (FCS; Sartorius *#*04-127-1A) in PBS, NPCs were re-suspended in Opti-Klear™ Live Cell Imaging Buffer (abcam ab27939). The fluorescence labeling was immediately measured by FACS and analyzed with FlowJO_v10.8.1 software. For this analysis, Live/Dead calcein violet-AM was utilized to gate the viable cells (calcein^+^), whereas the Mitochondrial Tracker Green fluorescence intensity served as a relative mitochondrial mass indicator. The mean fluorescence intensity of TMRE was calculated from the gated positive Live/Dead calcein violet-AM (calcein^+^) cells (Fig. 4A). In addition, we divided the cells into two types of populations, high ΔΨm cells (TMRE^high^/MitoTracker green^high^) and low ΔΨm cells (TMRE^low^/MitoTracker green^high^), using an arbitrary threshold that was applied similarly to all (see representative Fig. 4C). The relative percentage of cells with high ΔΨm, calculated as:

$$\frac{{(high\,{\Delta}{\Psi}m\,{{{{{{{\mathrm{\% }}}}}}}})}}{{(high\,{\Delta}{\Psi}m\,{{{{{{{\mathrm{\% }}}}}}}} + low\,{\Delta}{\Psi}m\,{{{{{{{\mathrm{\% }}}}}}}})}} \times 100$$

In order to visualize the quality of cell labeling utilizing the probes and reagents in this assay, we seeded 1 × 10^4^ cells (per well) in a PDL/laminin-coated black 96-well flat bottom plate and cultured them for 48 h at 37 °C with 5% CO_2_. The cells were washed once with PBS and then incubated with the probes’ mixture mentioned above for 20 min. Next, 100 µl Opti-Klear™ Live Cell Imaging Buffer was added to each well after washing with 5% FCS. The representative images were acquired using Nikon Eclipse Ti2 wide-field fluorescence microscope with 20× magnification (supplementary Fig. 3).

### Mitochondrial superoxide assay

The mitochondrial ROS (mROS) levels were evaluated utilizing MitoSOX^TM^ red mitochondrial superoxide (Invitrogen *#*M36008). The NPCs were detached and stained by Live/Dead calcein violet-AM (0.9 µM), MitoSOX (3 µM), and Mitochondrial Tracker Green (75.3 nM), similarly to the abovementioned ΔΨm assay protocol. In addition, we conducted a separate set of experiments with a higher concentration of 5.4 µM MitoSOX (Supplementary Figs. 5 and 7). The mROS levels were assessed by measuring the red fluorescence intensity of MitoSOX from the live (calcein^+^) NPCs population. In addition, we set an arbitrary threshold of Mitochondrial Tracker Green fluorescence intensity to gate out live (calcein^+^) NPCs with low mitochondrial mass. The data was acquired by FACS and analyzed with FlowJO_v10.8.1 software.

### Statistical analysis

Statistical analyses were performed using GraphPad Prism 9. Data were analyzed by parametric unpaired two-tailed t-tests and two-way ANOVA followed by posthoc Bonferroni’s corrected multiple comparisons. For all comparisons between groups or conditions, significance was set at *p*  <  0.05 using two-tailed t-tests or ANOVAs wherever necessary. Results are displayed as mean ± SEM in all graphs with the individual data points. Samples in which NPCs’ population was lower than 95% were excluded from all analyses. Sample sizes were chosen based on previous experience with these experiments of us and others, as cited throughout the manuscript.

## Results

### Cultured cells from the brains of E16.5 embryos entail similar populations in AS and WT littermates, the vast majority being neural precursor cells

In order to investigate mitochondrial oxidative stress and apoptosis in AS mice brains during late embryonic stage, we generated primary brain-derived cell cultures from WT and AS E16.5 fetal mice. Brain-derived cells were cultured with a complete embryonic NeuroCult™ Proliferation medium in EGF presence to maintain their self-renewal, proliferation, and differentiation potential (supplementary Fig. 1). At first, knowing that the paternal allele of *Ube3a* is still expressed during the embryonic period, we aimed to determine the expression levels of UBE3A protein in AS and WT brain-derived cultured cells at E16.5. Western blot analysis showed that UBE3A expression in the AS was reduced to 50% compared to WT (*t*_(6)_ = 5.68, *p* < 0.01 in t-test) (Fig. 1A).

To examine the efficiency of the culturing design in preserving the stemness entity, we labeled the cells with the neural precursor cells (NPCs) marker Nestin using FACS. As expected for this protocol, more than 98% of the cells were defined as Nestin^+^ cells, granting their validation as undifferentiated NPCs (Fig. 1B). The percentages of Nestin^+^ cells were similar between AS and WT NPCs (*t*_(10)_ = 0.04, *p* = 0.97 in t-test). Showing that the vast majority of cells expressed NPCs marker was further confirmed with fluorescence microscopy after immunostaining against Nestin (Fig. 1C). In addition, we used the FACS analysis for measuring BrdU incorporation following the application of a 45 min of BrdU pulse. This analysis showed a high percentage of BrdU^+^ NPCs (over 39%), with no significant difference between the genotypes (*t*_(10)_ = 1.19, *p* = 0.26 in t-test) (Fig. 1D). This indicates a high proliferation capacity, which is also a hallmark of NPCs. Altogether, we concluded that the populations of cells cultured from AS and WT embryonic brains are practically similar and homogenous, with almost all cells being NPCs from neuroectodermal origin.

### AS NPCs show an unaltered proliferation rate

**Fig. 2:**
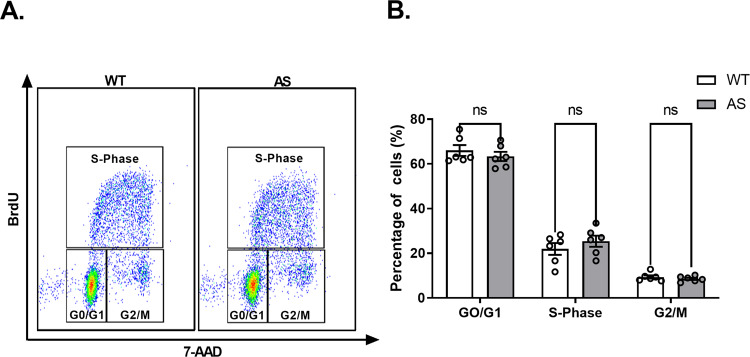
NPCs cultured in NeuroCult™ Proliferation medium display similar proportions of cell-cycle phases between AS and WT NPCs. **A** Representative dual-parameter FACS scatter plots of BrdU versus 7-AAD in WT and AS cultured NPCs. **B** WT and AS NPCs show a comparable percentage of cells at each cell-cycle stage. *N* = 6 for each group. Data are presented as means ± SEM (ns= non-significant in a two-way ANOVA).

Previously, we reported that *Ube3a* regulates essential cellular processes, such as proliferation and apoptosis, in full knockout *Ube3a* (*Ube3a*^p−/m−^) mouse embryonic fibroblasts (MEFs) [29]. Therefore, we first investigated the proliferation rate in AS NPCs using the Phase-Flow™ BrdU Cell proliferation Kit. This approach examines proliferation based on cell cycle progression, which is determined by incorporating BrdU into newly synthesized DNA versus the DNA content (7-AAD) (Fig. 2A). AS NPCs showed a comparable proportion of cell cycle phases to WT NPCs (*F*_(2,30)_ = 1.23, *p* = 0.31 for the interaction of genotype by cell cycle phase, and *F*_(1,30)_ = 8.418e-005, *p* = 0.99 for genotype effect in a two-way ANOVA) (Fig. 2B). Moreover, the percentage of cells in the DNA synthesis phase (S-phase), which precedes the distribution to two daughter cells, was similar between AS NPCs and WT NPCs (*t*_(30)_ = 1.23, *p* = 0.68 in posthoc Bonferroni corrected comparisons). Therefore, the results indicate that AS NPCs display a similar proliferation rate to WT NPCs.

### AS NPCs show enhanced apoptosis

**Fig. 3:**
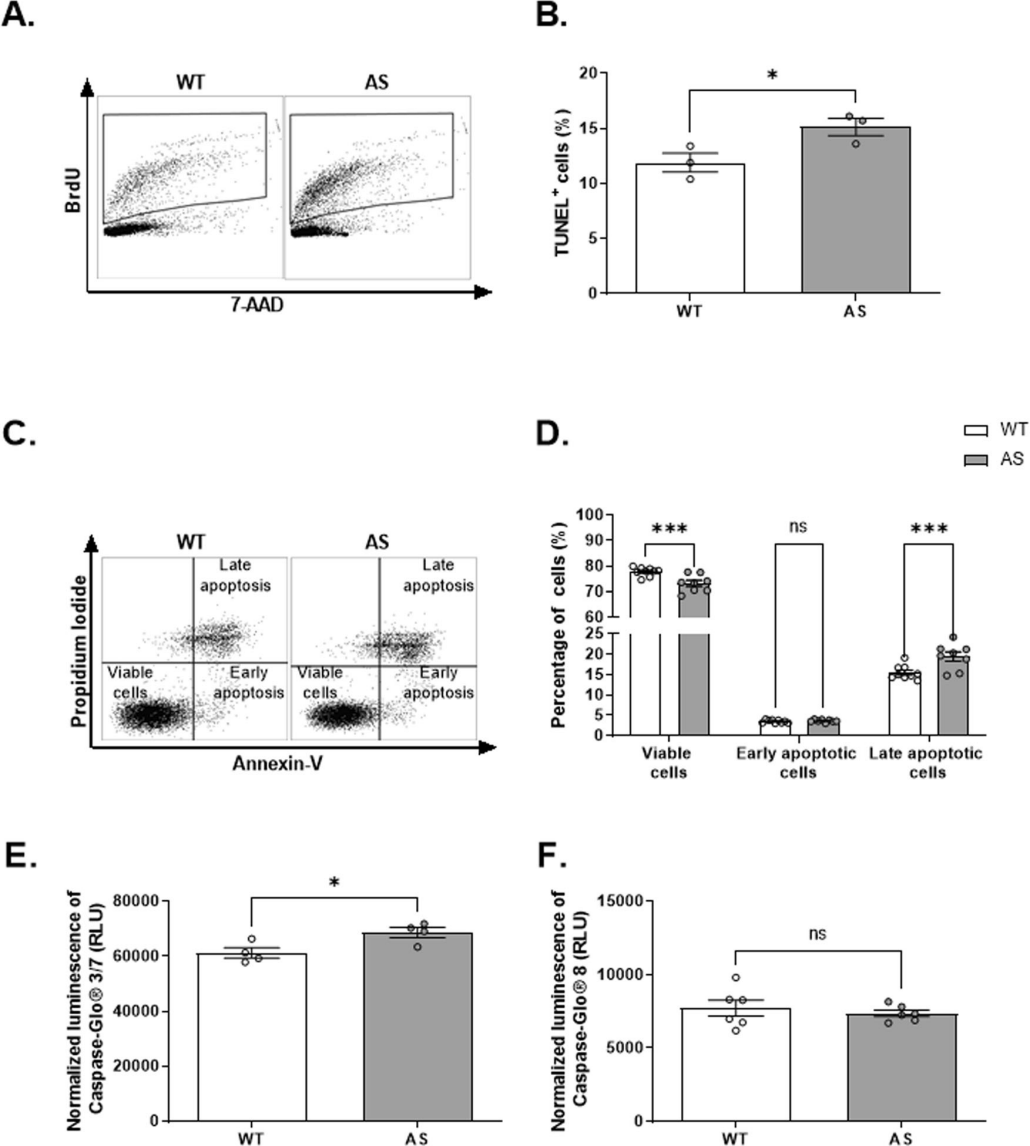
AS NPCs exhibit enhanced apoptosis. **A** Representative FACS scatter plots of TUNEL assay measuring the percentage of apoptosis in WT and AS cultured NPCs. **B** AS show a significantly higher percentage of TUNEL-positive (TUNEL^+^) cells than WT NPCs. *N*  =  3 for each group. **C** Representative dual-parameter FACS scatter plots of Annexin-V versus propidium iodide (PI) measure the apoptosis percentage in WT and AS cultured NPCs. **D** AS NPCs show a higher percentage of late apoptotic cells and a lower percentage of viable cells compared to WT NPCs, as measured by Annexin-V/PI dual-staining. *N*  =  9 and *N*  =  8 for WT and AS NPCs, respectively. **E** AS NPCs show increased caspase-3/7 enzymatic activity compared to WT NPCs. Caspase-3/7 luminescence is normalized to CellTiter 96® AQueous One Solution Cell Proliferation (MTS) absorbance. *N* = 4 for each group. From here on, RLU denotes relative light units. **F** WT and AS NPCs show comparable caspase-8 enzymatic activity. Caspase-8 luminescence is normalized to MTS absorbance. *N* = 6 for each group. For all panels, data are presented in means ± SEM (for **B**, **E**, **F**: ns= non-significant and *p < 0.05 in t-test; for **D**: ns = non-significant and ***p < 0.001 in two-way ANOVA).

Next, we investigated the apoptosis rate of AS NPCs by quantifying the late apoptotic cells associated with DNA fragmentation utilizing the TUNEL assay (Fig. 3A-B). AS NPCs exhibited enhanced apoptosis, as demonstrated by a significantly higher percentage of TUNEL^+^ NPCs in AS compared to WT (*t*_*(*4)_ = 2.78, *p* < 0.05 in t-test). To further confirm the increased apoptosis in AS NPCs, we next performed the Annexin-V/PI assay (Fig. 3C, D), which also allows for distinguishing between viable and apoptotic cells. Coinciding with the above finding, we found significant differences in cell viability between the genotypes (*F*_(2,45)_ = 17.89, p < 0.0001 for the interaction of genotype by cell subpopulation in two-way ANOVA) (Fig. 3D). AS NPCs displayed significantly more late apoptotic cells, concomitant with a significantly reduced percentage of viable cells compared to WT NPCs (*t*_(45)_ = 3.89, *p* = 0.001 and *t*_(45)_ = 4.55, *p* = 0.0001, respectively in posthoc Bonferroni corrected comparisons). The portion of early apoptotic cells was not significantly differed between AS and WT NPCs (*t*_(45)_ = 0.14, *p* > 0.99 in posthoc Bonferroni corrected comparisons).

Apoptosis evolves by sequential activation of caspases by proteolytic cleavage, culminating in the activation of caspase-3 and 7 (caspase-3/7) [40]. Therefore, we used the measurement of caspase-3/7 activity as a proxy to assess apoptosis. To account for variations in cell number, we normalized the caspase-3/7 and all the other luminescence-based measurements to the relative number of cells by MTS assay. It is critical to note that the relative cell numbers were comparable between AS and WT NPCs (*t*_(6)_ = 0.25, *p* = 0.81 in t-test) (Supplementary Fig. 4). AS NPCs showed elevated levels of caspase-3/7 activity compared to WT NPCs (*t*_(6)_ = 2.86, *p* < 0.05 in t-test), also indicating enhanced apoptosis in AS (Fig. 3E). Caspase-3/7 activation is the final common pathway of both the extrinsic and the intrinsic apoptotic pathways [40]. Therefore, it raises the question of which pathway is more activated in AS NPCs. To address this, we measured the activity of caspase-8, the initiator of the extrinsic pathway, which showed no differences between AS and WT NPCs (*t*_(10)_ = 0.6, *p* = 0.56 in t-test) (Fig. 3F). This suggests that the enhancement of apoptosis in AS NPCs is induced via the intrinsic apoptotic signaling pathway and not via the extrinsic pathway.

### AS NPCs show enhanced mitochondrial membrane potential

**Fig. 4:**
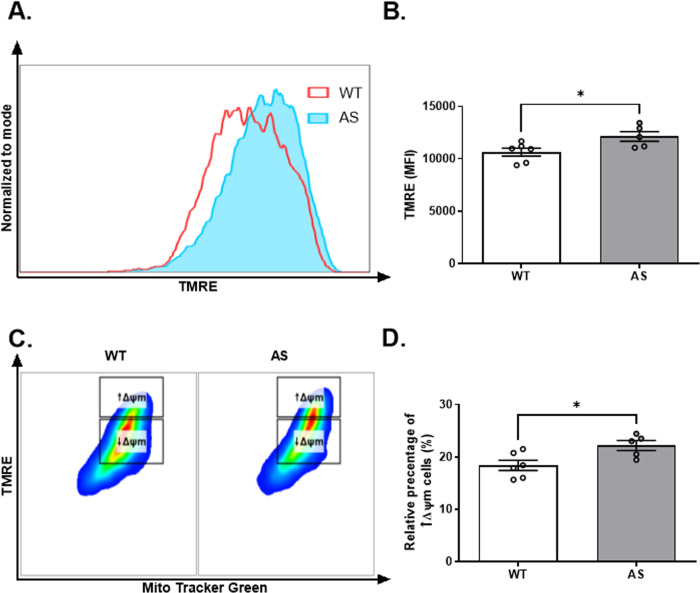
Mitochondrial membrane potential (ΔΨm) is higher in AS NPCs. **A** A representative FACS-derived histogram of the TMRE fluorescence intensity, measuring the ΔΨm of WT and AS cultured NPCs. **B** AS show enhanced ΔΨm compared to WT NPCs, as defined by the TMRE-mean fluorescence intensity (MFI). **C** Representative dual-parameter FACS plots of Mito Tracker Green and TMRE. The rectangular boxes represent the two populations which were arbitrarily defined as NPCs with high (↑ΔΨm) and low ΔΨm (↓ΔΨm). **D** AS consist a higher percentage of cells with high ΔΨm compared to WT NPCs. *N*  =  6 and *N*  =  5 for WT and AS, respectively. For all panels, data are presented in means ± SEM (*p < 0.05 in t-test).

The mitochondria are the functional hub of the intrinsic apoptotic pathway, thus essential for cell survival and function [40]. This mitochondrial regulation is affected by several mitochondrial functions, such as ATP and ROS production [41], which are firmly dependent on the mitochondrial membrane potential (ΔΨm) [42]. To measure the ΔΨm in living cells, we utilized the TMRE assay, as previously described [39]. AS NPCs showed a higher TMRE mean fluorescence intensity compared to the WT NPCs (*t*_(9)_ = 2.56, *p* < 0.05 in t-test) (Fig. 4A, B). Since lower mitochondrial content might hinder the mitochondrial TMRE accumulation, we further analyzed by setting a threshold to the mitochondrial mass indicator- MitoTracker green [39]. Within this population of cells with relatively high mitochondrial mass (MitoTracker green^high^), we arbitrarily defined two subpopulations of cells: one with high ΔΨm (TMRE^high^/MitoTracker green^high^) while the other is manifested by low ΔΨm (TMRE^low^/MitoTracker green^high^) (Fig. 4C). The relative percentage of cells with high ΔΨm was significantly higher in AS NPCs than in WT NPCs (*t*_(9)_ = 2.78, *p* < 0.05 in t-test) (Fig. 4D). Therefore, these findings indicate a more hyperpolarized ΔΨm in AS NPCs compared to WT NPCs.

### AS NPCs show enhanced mROS levels

**Fig. 5:**
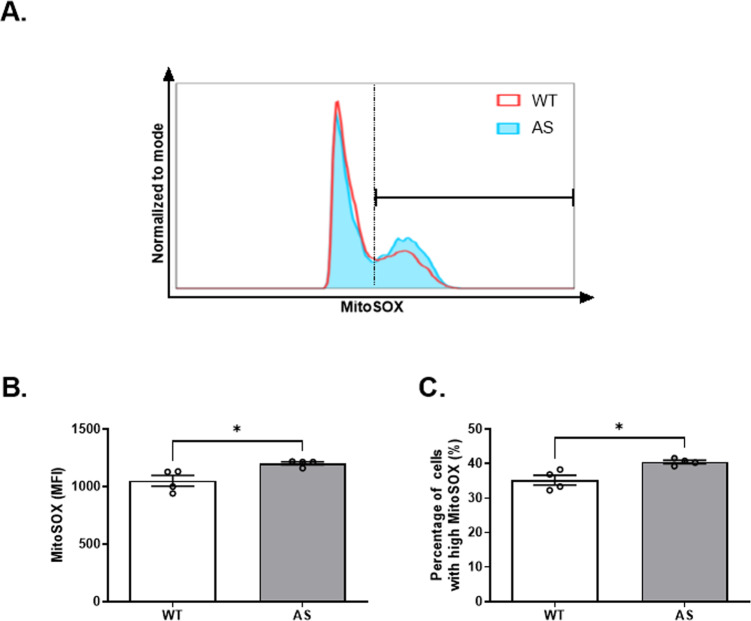
Elevated levels of mitochondrial superoxide in AS NPCs. **A** A Representative FACS-derived histogram of the MitoSOX fluorescence intensity, measuring the mitochondrial superoxide levels of WT and AS cultured NPCs. The dashed vertical line depicts the threshold for “high” MitoSOX fluorescence (see **C**). **B** AS NPCs show a higher mean fluorescence intensity (MFI) of MitoSOX than WT NPCs. **C** AS NPCs consist a higher percentage of cells with high fluorescence intensity of MitoSOX. *N*  =  4 for each group. For all panels, data are presented in means ± SEM (*p < 0.05 in t-test).

The ΔΨm and mROS production are reciprocally linked [42–44]. Alterations in ΔΨm affect mROS production and vice versa. Given the elevation of ΔΨm in AS NPCs, we turned to assess mROS levels using MitoSOX staining (Fig. 5A). Since concerns have recently been raised regarding the reliability of the conventionally used concentration (5 μM MitoSOX) to detect the mitochondrial superoxide solely [45], we utilized a lower concentration of 3 μM. AS NPCs demonstrated overall excessive mROS levels compared to WT NPCs, as determined by the significantly higher MitoSOX mean fluorescence intensity (*t*_(6)_ = 3.07, *p* < 0.05 in t-test) (Fig. 5B). Coinciding, as evident from the representative histogram (Fig. 5A), AS showed a higher percentage of cells with high MitoSOX fluorescence intensity compared to WT NPCs (*t*_(6)_ = 3.52, *p* < 0.05) (Fig. 5C). Of note, we obtained corresponding high mROS levels in AS NPCs also while using the higher concentration of 5.4 μM MitoSOX (*t*_(14)_ = 2.73, *p* < 0.05 and *t*_(14)_ = 2.5, *p* < 0.05 for MitoSOX mean fluorescence intensity and cell percentage with high MitoSOX, respectively in t-test) (Supplementary Fig. 5). Taken together, MitoSOX experiments show higher levels of mROS in AS NPCs.

### AS NPCs display reduced glutathione levels

**Fig. 6:**
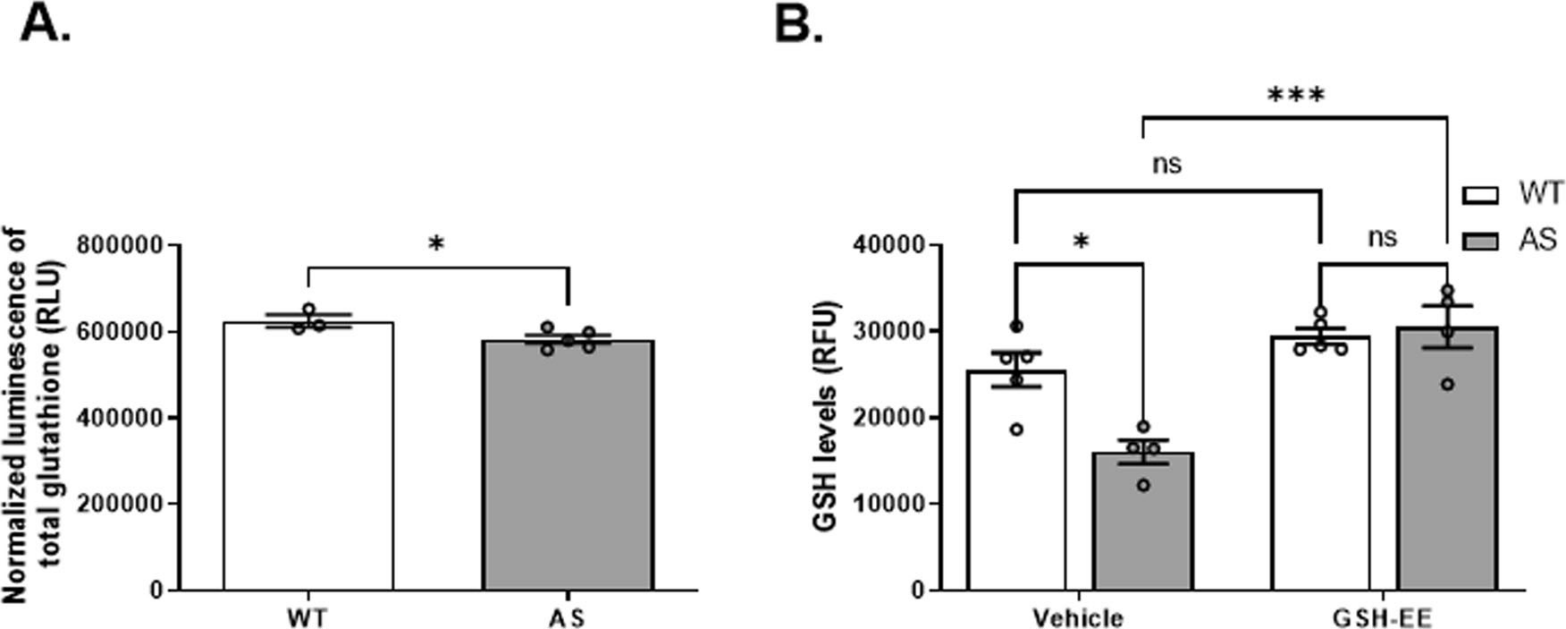
Glutathione-reduced ethyl ester (GSH-EE) treatment replenishes the low GSH levels in AS NPCs. **A** AS NPCs show lower total glutathione levels than WT NPCs. The glutathione luminescence signal is normalized to MTS absorbance. *N*  =  3 and *N*  =  5 for WT and AS NPCs, respectively. **B** AS NPCs show markedly lower levels of reduced glutathione (GSH), which were significantly restored following 48 h treatment with 0.5 mM GSH-EE. *N*  =  5 and *N*  =  4 for WT and AS NPCs, respectively. Data are presented in means ± SEM (for **A**: *p < 0.05 in t-test, and for **B**: ns= non-significant, *p < 0.05, ***p < 0.001 in two-way ANOVA).

Enhanced accumulation of mROS may derive from either excessive ROS formation or limited activity of the compensatory antioxidant machinery. One of the most abundant endogenous antioxidants in the brain is glutathione which counteracts the mROS [46, 47]. Therefore, we examined the total glutathione levels, comprising the oxidized glutathione (GSSG) and the reduced glutathione (GSH) forms. AS NPCs showed significantly lower total glutathione levels compared to WT NPCs (*t*_(6)_ = 2.53, *p* < 0.05 in t-test) (Fig. 6A). Furthermore, we found that the basal levels of GSH in AS NPCs were 37% lower than in WT NPCs (*t*_(14)_ = 3.86, *p* < 0.05 in posthoc Bonferroni corrected comparisons) (Fig. 6B).

The indispensable reducing agent of glutathione, NADPH, is tightly coupled to the glutathione system for protection against oxidative stress [48, 49]. Therefore, we next evaluated the NADPH and NADP^+^ levels. In line with the reduced GSH levels, we obtained significantly lower NADPH levels in AS compared to WT NPCs (*t*_(6)_ = 2.55, *p* < 0.05 in t-test) (Supplementary Fig. 6A). In contrast, NADP^+^ levels were comparable between the genotypes (*t*_(6)_ = 0.21, *p* = 0.84 in t-test) (Supplementary Fig. 6B). These data suggest that the elevated mROS levels in AS could be, at least in part, due to accumulation as a result of a flawed glutathione-mediated antioxidant system.

### Replenishment of GSH levels subsides the excessive mROS levels but not the higher ΔΨm in AS NPCs

To better correlate the consequences of GSH deficiency to the excessive mROS levels in AS NPCs, we treated the cells with GSH-EE, a cell-permeable GSH supplement [46, 50]. Treatment with 0.5 mM GSH-EE for 48 h effectively replenished the GSH levels of AS NPCs (*F*_(1,14)_ = 9.21, *p* < 0.01 for the interaction of genotype by treatment; *F*_(1,14)_ = 5.9, *p* < 0.05 and *F*_(1,14)_ = 27.94, *p* < 0.001 for genotype and treatment effects in a two-way ANOVA, respectively) (Fig. 6B). GSH-EE administration significantly increased the GSH levels of AS NPCs compared to their basal levels (*t*_(14)_ = 5.58, *p* < 0.001) and restored the GSH levels to the WT-treated NPCs levels (*t*_(14)_ = 0.43, *p* > 0.99 in posthoc Bonferroni corrected comparisons).

**Fig. 7:**
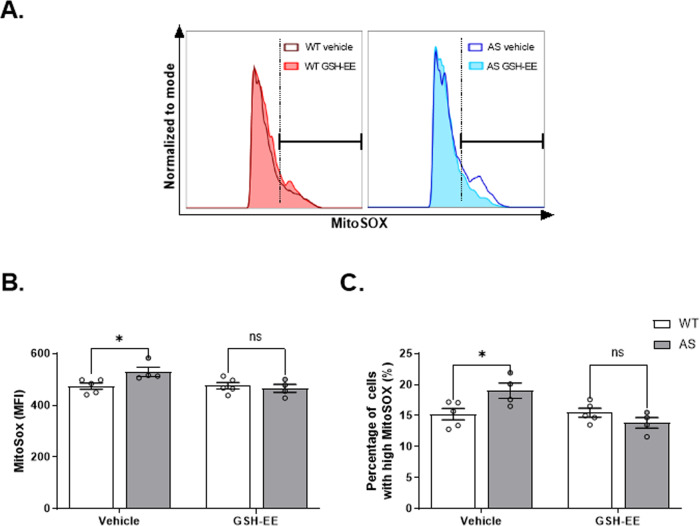
GSH replenishment by GSH-EE application reduced the excessive mROS levels of AS NPCs to WT levles. **A** Representative FACS-derived histograms of MitoSOX fluorescence intensity of WT and AS for vehicle and GSH-EE-treated NPCs. The dashed vertical line depicts the threshold for “high” MitoSOX fluorescence (see **C**). **B** GSH-EE treatment mitigates the excessive mitochondrial superoxide levels of AS NPCs to the WT-treated NPCs levels, as defined by the mean intensity (MFI) signal of MitoSOX. **C** GSH-EE treatment reduces the increased percentage of cells with high MitoSOX fluorescence intensity observed in AS NPCs to comparable percentage of the WT NPCs. *N*  =  5 and *N*  =  4 for WT and AS NPCs, respectively. For all panels, data are presented in means ± SEM (ns= non-significant, *p < 0.05 in two-way ANOVA).

Having established the efficacy of GSH-EE treatment in replenishing the GSH levels in AS NPCs, we examined whether it further attenuates the excessive mROS levels in AS NPCs. Indeed, GSH-EE treatment reduced the mROS levels in AS NPCs, as measured by mean fluorescence intensity of MitoSOX (*F*_(1,14)_ = 5.14, *p* < 0.05 for the interaction of genotype by treatment; *F*_(1,14)_ = 2.37, *p* = 0.15 and *F*_(1,14)_ = 4.65, *p* < 0.05 for genotype and treatment effects in a two-way ANOVA, respectively). As opposed to the significantly higher MitoSOX mean fluorescence intensity of AS-vehicle NPCs compared to WT-vehicle NPCs (*t*_(14)_ = 2.69, *p* < 0.05), both genotypes displayed similar levels of MitoSOX following GSH-EE treatment (*t*_(14)_ = 0.51, *p* > 0.99 in posthoc Bonferroni corrected comparisons) (Fig. 7A, B). Also, the percentage of AS NPCs with high MitoSOX fluorescence intensity diminished remarkably in response to the treatment (*F*_(1,14)_ = 8.53, *p* < 0.05 for the interaction of genotype by treatment; *F*_(1,14)_ = 1.34, *p* = 0.27 and *F*_(1,14)_ = 7.2, *p* < 0.05 for genotype and treatment effects in a two-way ANOVA, respectively). AS-vehicle NPCs exhibited significantly higher percentage of cells with high MitoSOX levels compared to WT-vehicle NPCs (*t*_(14)_ = 2.88, *p* < 0.05), whereas GSH-EE treatment reduced the percentage of cells with high MitoSOX levels in AS treated NPCs to be comparable to that percentage in WT-treated NPCs (*t*_(14)_ = 1.25, *p* = 0.47 in posthoc Bonferroni corrected comparisons) (Fig. 7C). Likewise, when we repeated the same experiment with a higher concentration of 5.4 µM MitoSOX, we obtained a corresponding treatment effect of lowering MitoSOX mean fluorescence intensity (*F*_(1,8)_ = 14.5, *p* < 0.01 for interaction of genotype by treatment; *F*_(1,8)_ = 4.3, *p* = 0.07 and *F*_(1,8)_ = 10.49, *p* < 0.05 for treatment and genotype effects in a two-way ANOVA, respectively) as well as lowering the proportional fraction of cells with high MitoSOX fluorescence intensity (*F*_(1,8)_ = 11.49, *p* < 0.01 for interaction of genotype by treatment; *F*_(1,8)_ = 3.4, *p* = 0.1 and *F*_(1,8)_ = 9.96, *p* < 0.05 for genotype and treatment effects in a two-way ANOVA, respectively) (supplementary Fig. 7).

**Fig. 8:**
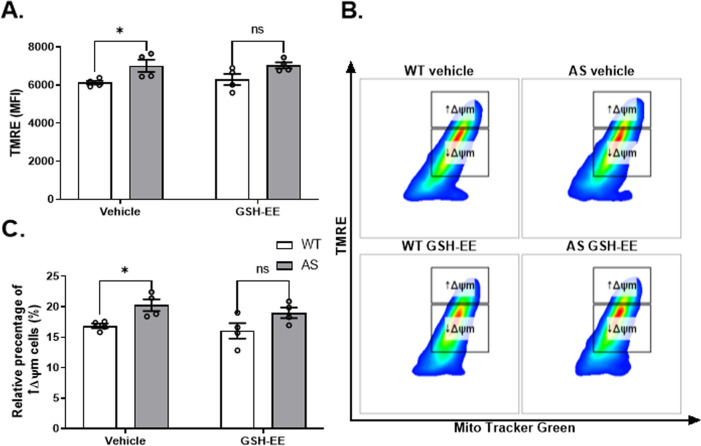
GSH replenishment by GSH-EE application does not restore the enhanced mitochondrial membrane potential (ΔΨm) in AS NPCs. **A** AS-vehicle NPCs show enhanced ΔΨm compared to WT-vehicle NPCs, as defined by the TMRE- mean fluorescence intensity (MFI). GSH-EE-treated AS and WT NPCs do not significantly differ in TMRE MFI, yet, the treatment effect does not reach significance. **B** Representative dual-parameter FACS plots of Mito Tracker Green and TMRE. The rectangular boxes represent the two populations which were arbitrarily defined as NPCs with high (↑ΔΨm) and low ΔΨm (↓ΔΨm). **C** Unlike AS-vehicle, AS-treated NPCs do not display a higher ratio of cells with high ΔΨm than WT-treated NPCs, yet, the treatment effect is insignificant. *N*  =  4 for each group. For all panels, data are presented in means ± SEM (ns= non-significant, *p < 0.05 in two-way ANOVA).

In contrast to GSH-EE repressive effect on mROS levels in AS NPCs, no significant treatment effect was found on ΔΨm, as assessed by TMRE mean fluorescence intensity (*F*_(1,12)_ = 0.09, *p* = 0.77 for the interaction of genotype by treatment; *F*_(1,12)_ = 11.76, *p* < 0.01 and *F*_(1,12)_ = 0.16, *p* = 0.7 for genotype and treatment effects in a two-way ANOVA, respectively) (Fig. 8A), and the relative percentage of cells with high ΔΨm (*F*_(1,12)_ = 0.05, *p* = 0.82 for the interaction of genotype by treatment; *F*_(1,12)_ = 11.6, *p* < 0.01 and *F*_(1,12)_ = 1.23, *p* = 0.29 for genotype and treatment effects in a two-way ANOVA, respectively) (Fig. 8B, C). Although posthoc Bonferroni corrected multiple comparisons show that the GSH-EE treatment diminished the ΔΨm of AS to comparable ΔΨm of the WT (*t*_(12)_ = 2.64, *p* < 0.05 and *t*_(12)_ = 2.57, *p* < 0.05 between AS-vehicle and WT-vehicle NPCs; and *t*_(12)_ = 2.21, *p* = 0.09; *t*_(12)_ = 2.24, *p* = 0.09 between AS-treated and WT-treated NPCs, for TMRE MFI and cell percentage with high ΔΨm, respectively), as if there was a treatment effect that evened the two genotypes (Fig. 8), taking a lesser strict statistical comparison (Fisher’s LSD test) still showed a significant genotype difference between the GSH-EE-treated groups (*t*_(12)_ = 2.21, *p* < 0.05 and *t*_(12)_ = 2.24, *p* < 0.05 between AS-treated and WT-treated NPCs, for TMRE MFI and cell percentage with high ΔΨm, respectively). Altogether, these findings indicate that the GSH-EE treatment might be beneficial in scavenging the excessive mROS in AS NPCs rather than restoring the ΔΨm aberration in this cell population.

### GSH replenishment attenuates the enhanced apoptosis in AS NPCs

**Fig. 9:**
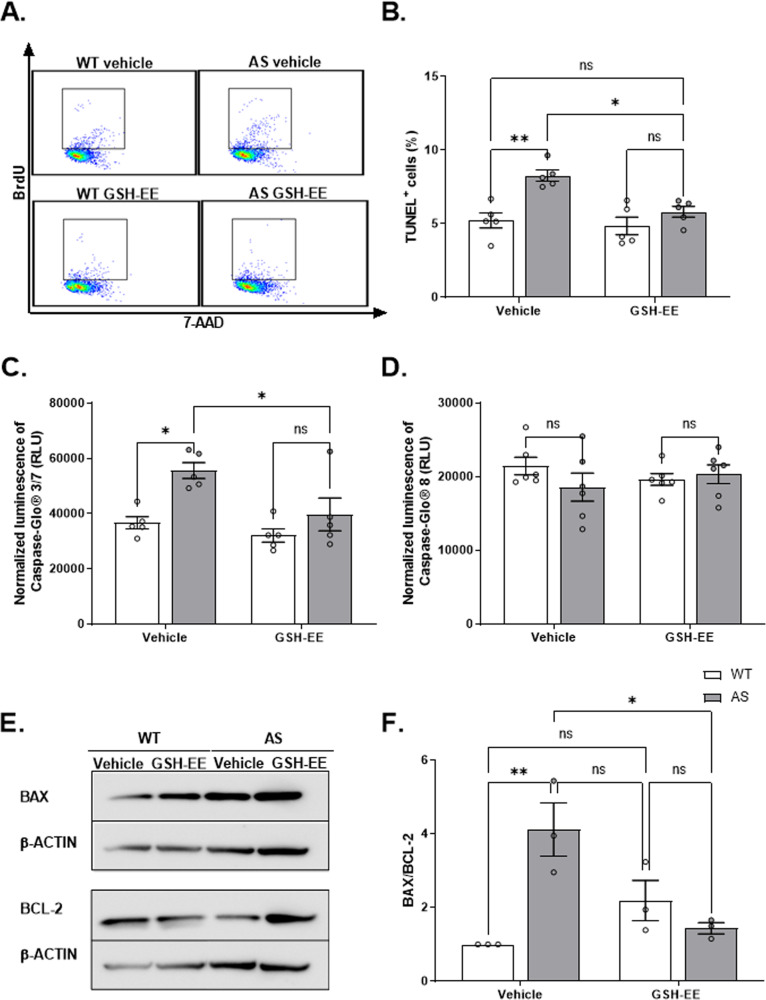
GSH replenishment by GSH-EE application attenuates the enhanced apoptosis in AS NPCs. **A** Representative dual-parameter FACS scatter plots of TUNEL assay measuring the percentage of apoptosis in WT and AS for vehicle and treated cultured NPCs. **B** GSH-EE treatment reduced the TUNEL^+^ cell percentage in AS-treated NPCs to WT-treated NPCs levels, thus effectively restraining the apoptosis in AS NPCs. *N*  =  5 for each group. **C** In contrast to vehicle groups, AS-treated NPCs and WT-treated NPCs exhibit similar levels of caspase-3/7 enzymatic activity. Caspase-3/7 luminescence is normalized to MTS absorbance. *N* = 5 for each group. **D** WT and AS NPCs show comparable caspase-8 enzymatic activity, which is not significantly affected by GSH-EE treatment. Caspase-8 luminescence is normalized to MTS absorbance. *N* = 6 for each group. **E** Representative western blots of the pro-apoptotic BAX protein, the anti-apoptotic BCL-2 protein, and the expression of β-ACTIN as a loading control. **F** The expression ratio of BAX/BCL-2 in AS NPCs is reduced to the ratio in WT-treated NPCs following GSH-EE treatment. All values are normalized to the β-ACTIN. Quantification was done as a ratio to WT-vehicle on the same blot. *N* = 3 for each group. For all panels, data are presented in means ± SEM (ns = non-significant, *p < 0.05 and **p < 0.01 in two-way ANOVA).

Given the contribution of GSH-EE in normalizing the mROS levels of AS NPCs, we utilized the TUNEL assay to investigate whether GSH-EE treatment imposes a protective effect that attenuates the enhanced apoptosis in AS NPCs. As expected, administration of GSH-EE mitigated the observed enhanced apoptosis in AS NPCs (*F*_(1,16)_ = 4.81, *p* < 0.05 for the interaction of genotype by treatment; *F*_(1,16)_ = 17.92, *p* < 0.001, and *F*_(1,16)_ = 8.99, *p* < 0.01 for genotype and treatment effects in two-way ANOVA, respectively) (Fig. 9A-B). Posthoc Bonferroni corrected comparisons showed that AS-vehicle NPCs displayed significantly higher TUNEL^+^ cell percentage compare to the WT-vehicle (*t*_(16)_ = 4.55, *p* < 0.01), while GSH-EE treatment moderated the apoptosis of AS NPCs to that of the WT-treated NPCs (*t*_(16)_ = 3.67, *p* < 0.05, between AS-vehicle to AS-treated, *t*_(16)_ = 1.44, *p* > 0.99, between AS-treated to WT-treated).

In addition, GSH replenishment decreased the activity of caspase-3/7, the primary executors of apoptosis, in AS-treated NPCs (*F*_(1,16)_ = 12.98, *p* < 0.01 and *F*_(1,16)_ = 7.69, *p* < 0.05, for genotype and treatment effects in a two-way ANOVA, respectively) (Fig. 9C). Posthoc Bonferroni comparisons show that while AS-vehicle NPCs showed a significantly enhanced activity of caspase-3/7 relative to WT-vehicle NPCs (*t*_(16)_ = 3.64, *p* < 0.05, between AS-vehicle to WT-vehicle), treating the AS NPCs with GSH-EE attenuated the activity of caspase-3/7 by 28% to the levels of the WT-treated NPCs (*t*_(16)_ = 3.05, *p* < 0.05, between AS-vehicle to AS-treated; *t*_(16)_ = 1.45, *p* = 0.99, between AS-treated to WT-treated).

We next assessed the activity of caspase-8, the initiator of the extrinsic apoptosis pathway, in response to GSH-EE treatment. Since we did not observe a difference in caspase-8 activity (Fig. 3F), it was not surprising that GSH-EE treatment had no effect on caspase-8 activity (*F*_(1,20)_ = 0.001, *p* = 0.98 for treatment effect in a two-way ANOVA) (Fig. 9D).

Since apoptosis and mitochondria are clearly affected in AS NPCs, we also examined whether GSH-EE treatment affected the expression ratio of two pivotal apoptosis regulators that act at the mitochondria, the pro-apoptotic BAX protein and the anti-apoptotic BCL-2 protein (BAX/BCL-2 ratio). GSH-EE treatment differentially affected BAX/BCL-2 ratio (F_(1,8)_ = 17.74, *p* < 0.01 for the interaction of genotype by treatment; *F*_(1,8)_ = 6.61, *p* < 0.05 for genotype effect in a two-way ANOVA) (Fig. 9E, F). Main treatment effect was insignificant (*F*_(1,8)_ = 2.62, *p* = 0.14 for the treatment effect in a two-way ANOVA). Posthoc Bonferroni corrected comparisons showed enhanced BAX/BCL-2 ratio in AS-vehicle NPCs compared to WT-vehicle (*t*_(8)_ = 4.8, *p* < 0.01), while GSH-EE treatment significantly decreased BAX/BCL-2 ratio in AS NPCs (*t*_(8)_ = 4.12, *p* < 0.05 between AS-vehicle and AS-treated) to comparable levels of WT-treated NPCs (*t*_(8)_ = 1.16, *p* > 0.99 between AS-treated and WT-treated). The results suggest that GSH replenishment attenuates the excessive mitochondrial mediated apoptotic pathway of AS NPCs.

### Depletion of intracellular GSH promotes apoptosis in WT NPCs

**Fig. 10:**
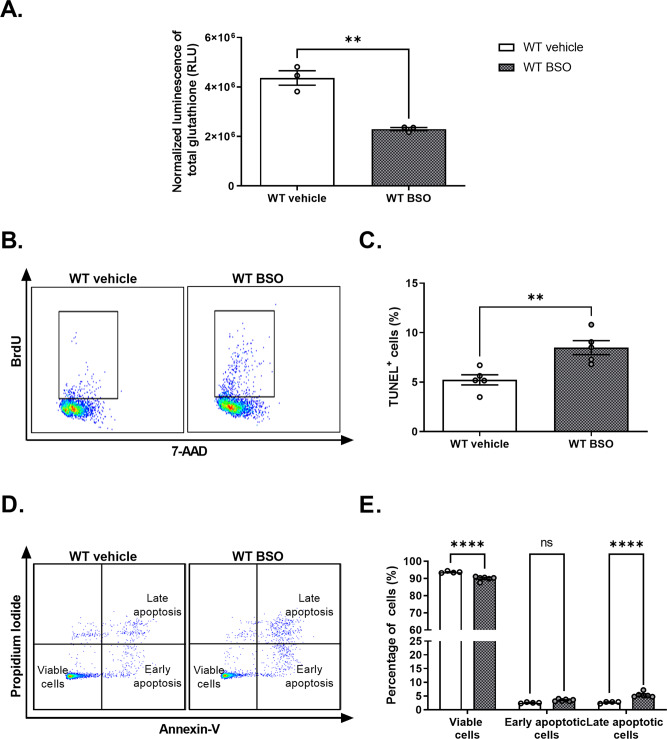
Glutathione depletion by L-Buthionine-sulfoximine (BSO) enhances apoptosis in WT NPCs. **A** WT NPCs show a significant reduction in total glutathione levels in response to treatment with 200 µM BSO for 5 h. WT luminescence signal is normalized to MTS absorbance. *N*  =  3 for each group. **B** Representative dual-parameter FACS scatter plots of TUNEL assay measuring the percentage of apoptosis in WT-vehicle NPCs and WT-BSO NPCs (20 h treatment with100 µM BSO). **C** WT-BSO NPCs exhibited a significantly higher percentage of TUNEL^+^ cells than WT-vehicle NPCs. *N* = 5 for each group. **D** Representative dual-parameter FACS scatter plots of Annexin-V versus propidium iodide (PI), measuring the percentage of apoptotic in WT-vehicle and WT-BSO treated NPCs (4 h treatment with 100 µM BSO). **E** WT-BSO NPCs show an increased percentage of late apoptotic cells and a reduced percentage of viable cells compared to WT-vehicle NPCs, as measured by Annexin-V/PI dual-staining. *N*  =  4 and *N*  =  6 for WT-vehicle and WT-BSO NPCs, respectively. Data are presented in means ± SEM (for **A** and **C**: **p < 0.01 in t-test, for panel E: ns=non-significant and ****p < 0.0001 in two-way ANOVA).

To further validate the involvement of low levels of glutathione in the induction of enhanced apoptosis in AS NPCs, we next evaluated whether glutathione depletion in WT NPCs may mimic the excessive apoptosis observed in AS NPCs. Treatment with 200 µM BSO for 5 h, significantly reduced the total glutathione levels compared to WT-vehicle NPCs (*t*_(4)_ = 6.9, *p* < 0.01 in t-test) (Fig. 10A). With this profound influence of BSO treatment on glutathione levels, we turn to investigate the apoptosis utilizing TUNEL and Annexin-V/PI assays. Treatment of 100 µM BSO for 20 h enhanced apoptosis in WT NPCs, as implied by a significant increase in TUNEL^+^ cell percentages (*t*_(8)_ = 3.72, *p* < 0.01 in t-test) (Fig. 10B-C). Moreover, a shorter incubation of only 4 h was sufficient to trigger late apoptosis in WT NPCs as demonstrated by Annexin-V/PI assay (*F*_(2,24)_ = 47.14, *p* < 0.0001 for the interaction of apoptotic phase by treatment in two-way ANOVA) (Fig. 10D-E). WT-BSO NPCs displayed a significant reduction in the percentage of viable cells compared to WT-vehicle NPCs (*t*_(24)_ = 7.7, *p* < 0.0001), together with a significant increase in late apoptotic cell percentage (*t*_(24)_ = 5.6, *p* < 0.0001 in posthoc Bonferroni corrected comparisons). These findings support the notion that alteration of glutathione levels in AS NPCs play a role in their vulnerability to apoptosis.

## Discussion

Mitochondrial dysfunctionalities have long been described in various health conditions such as cancer [51], neurodegenerative [52], and neurodevelopmental disorders [53]. The herein study shows multifaceted mitochondrial aberration in AS brain-derived NPCs generated from E16.5 mice. AS NPCs exhibit elevated ΔΨm, mROS, and apoptosis levels and a reduction in the GSH levels. Studying mitochondrial abnormalities in AS utilizing NPCs retrieved from the late embryonic stage provides a powerful avenue toward an innovative and broader view of AS pathogenesis, starting with early development.

Early brain development comprises critical cell processes like proliferation, outgrowth, migration, and apoptosis. All of which precede the definitive neural differentiation and underlying brain maturation and functionality [54, 55]. Perturbations during this initial yet indispensable milestone may lead to deleterious consequences on brain development. Moreover, *Ube3a* reinstatement studies recently highlighted the late embryonic stage as the sensitive period for AS pathogenesis and therapeutic interventions, especially with regard to the autistic behavioral phenotypes [25, 27]. Therefore, harnessing the power of studying embryonic NPCs as a model of this initial developmental stages may shed light on the dynamic early developmental trajectories of AS pathogenesis.

During embryonic development, the size of the NPCs pool is determined by the proliferative capacity, intricately coordinated with the apoptosis rate. Aberration in this delicate balance can culminate with cytoarchitecture anomalies in the mature brain [56, 57]. Previously, we showed enhanced proliferation in Ube3a-null MEFs (*Ube3a*^p−/m−^) carrying a complete homozygous deletion of *Ube3a* [29]. However, no significant difference was found in AS NPCs with *Ube3a* maternal deletion compared to WT NPCs (Fig. 2). In the current study, NPCs were cultured with a proliferative medium containing EGF to maintain the NPCs proliferation and self-renewal capacity. Thus, it might compensate for and conceal existing proliferation deviation between AS and WT NPCs. On the other hand, when we evaluated the apoptotic capacity of the AS NPCs, we found it to be significantly higher compared to the WT NPCs (Fig. 3).

Programmed cell death during embryonic brain development is a physiological process that is crucial to optimize the number of progenitor cells and eliminate misplaced or damaged cells that might definitively differentiate improperly [58–60]. Enhanced apoptosis of NPCs during early stages of neural development can result in structural and functional defects that may be translated later during development to microcephaly and intellectual disability. Interestingly, absolute or relative microcephaly is defined as one of the core phenotypic traits of AS, penetrant in more than 80% of AS individuals by the age of two years [61]. AS rodent models also resemble the overall decreased brain size and neuroanatomical alterations in AS-affected humans [62–64]. Although these aberrations were mainly confined to white matter loss, extensive apoptosis of NPCs was previously suggested as an additional cause for the microcephaly in AS [65]. It is critical to note that contrary to the enhanced apoptosis in AS NPCs, we observed reduced apoptosis in Ube3a-null MEFs. This discrepancy may be explained by either the differential impact that UBE3A has on different cell types, thus emphasizing the importance of specifying the implications of UBE3A deficiency per the various types of brain-derived cells, or by the difference in the expression levels of UBE3A between AS (*Ube3a*^p+/m−^) and *Ube3a-*null (*Ube3a*^p−/m−^) [29].

Apoptosis is executed by a complex cascade of events activating caspases in response to extrinsic death ligands or intrinsic-mitochondrial signals. The final common pathway of the caspases cascades is the activation of caspase-3/7, which is considered the point ‘of no return’ [40], was significantly higher in AS NPCs (Fig. 3E). However, the activation of the initiator of the extrinsic pathway, caspase-8, was similar between AS and WT NPCs (Fig. 3F), which suggests that the observed enhancement in apoptosis is mainly by the intrinsic-mitochondrial apoptotic induction. Various stimuli, including metabolic and mitochondrial oxidative stress, can initiate the intrinsic cell death pathway [66, 67]. Mitochondria are considered the mainstay of most ATP synthesis through oxidative phosphorylation. Furthermore, mitochondria are involved in a broad range of vital physiological processes beyond their role in energy production, such as calcium buffering, redox homeostasis, and apoptosis, mirroring their crucial impact on cell fate [14–19, 41]. Therefore, mitochondrial dysfunction has been proposed as the common denominator of various neurodevelopment disorders [53]. Emerging evidence suggest a putative link between UBE3A disruptions and a broad perspective of mitochondrial abnormalities [8–12, 68]. In accordance, morphological and functional alterations, such as the impaired activity of complex-III of the mitochondrial respiratory chain, were found in the brains of AS model mice [8, 10, 12], and restoring the expression of complexes III and IV by CoQ10 analog ameliorates motor coordination, obsessive-compulsive behavior, and anxiety deficits in AS mice [11]. These complexes that serve as proton pumps are essential components in generating the ΔΨm, which builds up the driving force for ATP production [42]. Herein, we demonstrated that AS NPCs exhibited a higher ΔΨm (Fig. 4). This ΔΨm elevation hints toward either a homeostatic compensation for some impairment in the AS mitochondrial respiratory chain or it can result from this impairment, ending up with the accumulation of unutilized hydrogen ions.

The aforementioned elements of the mitochondrial respiratory chain, the ΔΨm and ATP production, are tightly coupled to the oxidative phosphorylation process and its inevitable by-product mROS [69]. Previously, we showed increased levels of mROS in CA1 hippocampal neurons of mature AS model mice, which were implicated in the impaired long-term hippocampal potentiation (LTP) and contextual fear memory deficits of AS mice [12]. In addition, we also revealed transcriptomic perturbations of mitochondrial- and ROS-related pathways in Ube3a-null MEFs [30]. Coinciding with these observations, we found a robust increase of mROS in AS NPCs (Fig. 5). Although homeostatic ROS levels play a role in several physiological processes, such as proliferation and differentiation, an uncontrolled increase of mROS levels were shown to result in deleterious oxidative stress and apoptosis [21, 23, 70].

Since the developing brain requires high energy and oxygen consumption, it is highly susceptible to oxidative stress [71]. The net mROS levels are tightly regulated by balancing ROS production and antioxidant-protective machinery [15]. The primary ROS scavenging system in the brain, the glutathione redox machinery that produces the antioxidant GSH, is inversely correlated with mROS accumulation [72, 73]. Interestingly, our previous transcriptomic analyses indicated glutathione dysregulation in the absence of UBE3A [30]. In the herein study we found lower levels of total glutathione and GSH in AS NPCs compared to NPCs extracted from WT littermates (Fig. 6). Furthermore, NADPH, the primary reductive agent of GSH, was significantly lower in AS NPCs than in WT NPCs (supplementary Fig. 6), which coincides with the excess mROS accumulation in AS NPCs. In principle, NADPH is required to enable glutathione reduction, which in turn is harnessed to mROS scavenging [46]. Based on all of the above, we hypothesized that the dysregulation of the glutathione redox system led to the elevated mROS levels in the AS NPCs.

Mitochondria are the primary intracellular sources of ROS, and both higher mitochondrial membrane potential and alterations in the electron transport chain can increase ROS production [74]. GSH exists as separate pools in the various cell compartments, including cytosol, nuclei, and mitochondria [75]. GSH synthesis does not occur in mitochondria [76], transport from the cytosol into the mitochondrial matrix is the principal mechanism that sustains mitochondrial GSH (mtGSH) levels. Since previous studies reported aberrant mitochondrial morphology in AS [10], and we previously observed that *Ube3a*-null MEFs exhibit altered mitochondria-associated pathways [29, 30], we posited that the AS mitochondrial abnormalities culminate in a reduced capacity of the mtGSH to control mROS accumulation. Membrane-permeant GSH derivatives, such as GSH-EE, have been found to increase cellular GSH in various tissues, including neuronal cell culture [77]. GSH-EE can lead to an increased level of mtGSH [78]. Therefore, to test the assumption that GSH replenishment will rescue some of the AS cellular phenotypes, we supplemented the AS and the WT NPCs cell culture with GSH-EE, and first showed that GSH-EE increased the GSH levels in AS NPCs to normal WT NPCs levels, but did not significantly enhanced GSH levels in WT NPCs (Fig. 6B). Next, we demonstrated that GSH-EE treatment reduced the mitochondrial superoxide levels in the AS NPCs and normalized the mitochondrial superoxide levels to both the WT-vehicle NPCs and the WT-treated NPCs levels (Fig. 7). Finally, we showed that GSH-EE reduced the apoptosis in AS NPCs, as it reduced and normalized the percentage of TUNEL^+^ cells (Fig. 9A-B), the activity of caspase-3/7 (Fig. 9C), and the BAX/BCL-2 ratio (Fig. 9E-F). Moreover, showing that caspase-8 was unaltered, and that GSH-EE did not affect caspase-8 activity (Fig. 9D), suggests that it is mainly the intrinsic apoptotic pathway that was involved and inhibited by GSH-EE supplementation [79]. These findings coincide with previous studies, which reported that intracellular GSH depletion is a critical determinant of apoptosis due to the resulting deleterious insults or perturbations to the mitochondrial redox environment [47].

Moreover, in an effort to recapitulate an AS-like state in WT NPCs, we supplemented the WT NPCs cell culture with BSO, a potent inhibitor of glutathione synthesis, which reduced the glutathione levels by ~50% (Fig. 10A). The reduction in the total glutathione levels led to a significant increase in the apoptosis of the WT NPCs (Fig. 10B-E), which corroborates the reduced GSH levels in AS NPCs (Fig. 6B) and their elevated apoptotic capacity (Fig. 3).

Noteworthy, despite the fact that replenishing the GSH levels decreased the excessive mROS levels and restored normal apoptosis levels, it did not rescue the elevated ΔΨm in AS NPCs (Fig. 8). Those findings imply that while the reduced GSH levels in AS NPCs contribute to the elevated mROS, the aberrant ΔΨm levels may derive from other AS mitochondria-related factors, such as structural aberrations and malfunction of the inner mitochondrial membrane or its respiratory complexes. Such aberrations may carry additional consequences, since ΔΨm plays a crucial role in mitochondrial homeostasis and mitochondrial functioning beyond ATP and ROS production. ΔΨm is the driving force for the transport of various molecules and ions other than protons [42], and proteins [42] necessary for optimal mitochondrial functioning in a healthy cell. For example, the mitochondria utilize ΔΨm for calcium buffering, and indeed, we have previously shown that calcium signaling is altered in various cellular models of AS, including brains of AS model mice [80].

To the best of our knowledge, this is the first study that addresses the involvement of UBE3A in regulating mitochondrial homeostasis during AS late embryonic development. Furthermore, herein we demonstrate enhanced apoptosis in embryonic brain cells of AS mice, which coincide with a glutathione-mediated redox imbalance and excessive mROS levels. Moreover, manipulation that increases the antioxidant GSH levels in the AS NPCs normalizes the mROS levels and rescues the apoptotic cellular phenotype of AS NPCs. These findings offer the first indication of glutathione involvement in the excessive mROS during the early stages of AS pathogenesis. In general, glutathione-mediated redox imbalance, either in terms of reductive stress or oxidative stress, may lead to dysregulation of the embryonic stem cell fate [81]. Thus, it may play a role in the pathogenesis of not only AS, but also in a range of neurodevelopmental disorders like fragile X syndrome, Rett syndrome, and other autistic disorders in which mitochondrial dysfunction and oxidative stress were shown to be involved [82–85]. Further studies are warranted to elucidate the relation between the biochemical mechanisms governed by UBE3A and the redox imbalance, and a more comprehensive understanding of the mechanism responsible for enhanced mitochondrial membrane potential during early brain ontogenesis of AS. This knowledge will pave the way toward novel therapeutic approaches addressing mitochondrial-related anomalies in early brain development, not only of AS pathogenesis but also other neurodevelopmental disorders.

### Supplementary information

Supplementary Material
