# Supplementary material for: Elevated ROS levels during the early development of Angelman syndrome alter the apoptotic capacity of the developing neural precursor cells
Source: Mol Psychiatry. 2023 Mar 29;28(6):2382–97. doi: 10.1038/s41380-023-02038-7 (PMC10611580; doi:10.1038/s41380-023-02038-7)
Supplement: Supplementary file 1 — Supplementary Material [file 41380_2023_2038_MOESM1_ESM.docx]

**Supplementary Information**


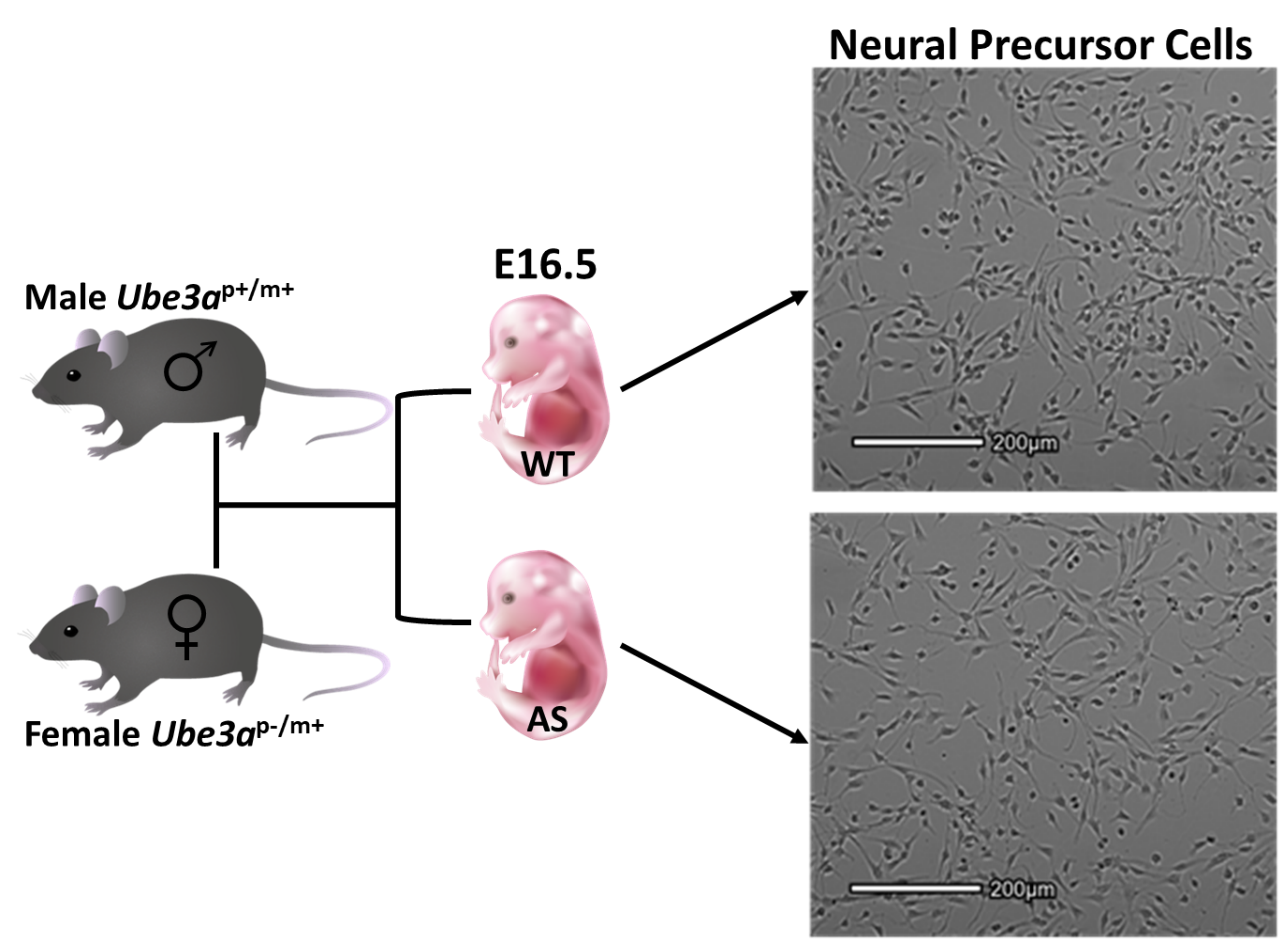


**Supplementary Figure 1. An illustration of WT and AS NPCs culture generation from E16.5 mouse brain embryos.** The representative bright-field images of neural precursor cells were acquired utilizing CytoSMART Omni FL system. Scale bar, 200 µm.

**Supplementary Figure 2. Representative images of apoptotic cells in WT and AS NPCs.** Apoptotic cells are labeled with propidium iodide (PI). The images were acquired utilizing the CytoSMART Omni FL system. Scale bar, 200 µm. The AS scan shows a higher number of PI-stained NPCs compared to the scan of WT NPCs.

**Supplementary Figure 3. Representative images visualize the mitochondrial membrane potential (ΔΨm) assay labeling in NPCs.** The images were acquired utilizing Nikon Eclipse Ti2 wide-field fluorescence microscope with 20x magnification. Scale bar, 50 µm.


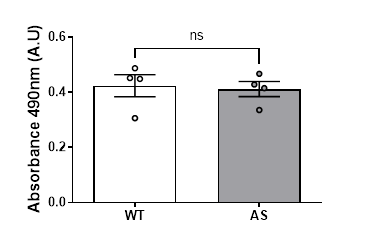


**Supplementary Figure 4. CellTiter 96® AQueous One Solution Cell Proliferation (MTS) absorbance is comparable between WT and AS NPCs.** The assay was used as a proxy to normalize the cell numbers. N = 4 for each group. A.U denotes arbitrary units. The data are presented in means ± SEM (ns= non-significant in t-test).

**Supplementary Figure 5. Elevated levels of mitochondrial superoxide in AS NPCs.** (A) A Representative FACS-derived histogram of the MitoSOX fluorescence intensity using 5.4µM of MitoSOX for measuring the mitochondrial superoxide levels of WT and AS cultured NPCs. The dashed vertical line depicts the threshold for "high" MitoSOX fluorescence (see panel C). (B) AS NPCs show higher mean mitochondrial superoxide levels than WT NPCs, as defined by the mean fluorescence intensity (MFI) of MitoSOX. (C) AS NPCs show a higher percentage of cells with high intensity of MitoSOX fluorescence. This figure shows similar results corresponding to the same experiment using 3µM of MitoSOX as in Figure 5. N = 8 for each group. For all panels, data are presented in means ± SEM (*p<0.05 in t-test).

**Supplementary Figure 6. AS NPCs display reduced NADPH levels.** (A) AS NPCs show lower NADPH levels compared to WT NPCs. NADPH luminescence is normalized to MTS absorbance. (B) WT and AS NPCs show similar NADP^+^ levels. NADP^+^ luminescence is normalized to MTS absorbance. N = 4 for each group. For all panels, data are presented in means ± SEM (ns= non-significant, *p<0.05 in t-test).

**Supplementary Figure 7. GSH replenishment by GSH-EE application reduces the excessive mROS levels of AS NPCs to WT levels.** (A) Representative FACS-derived histograms of MitoSOX fluorescence intensity of WT and AS for vehicle and GSH-EE treated NPCs, utilizing 5.4µM of MitoSOX. The dashed vertical line depicts the threshold for "high" MitoSOX fluorescence (see panel C). (B) GSH-EE treatment mitigates the excessive mitochondrial superoxide levels of AS NPCs to the WT-treated NPCs levels, as defined by the mean intensity signal of MitoSOX. (C) GSH-EE treatment reduces the increased percentage of cells with high MitoSOX fluorescence intensity levels in AS NPCs to comparable percentage of the WT NPCs. This figure shows similar results corresponding to the same experiment using 3µM of MitoSOX as in Figure 7. N = 3 for each group. For all panels, data are presented in means ± SEM (ns= non-significant, *p<0.05 and **p<0.01in two-way ANOVA).
